# Comparison of (Cost-)Effectiveness of Magnetic Resonance Image–Guided High-Intensity–Focused Ultrasound With Standard (Minimally) Invasive Fibroid Treatments: Protocol for a Multicenter Randomized Controlled Trial (MYCHOICE)

**DOI:** 10.2196/29467

**Published:** 2021-11-24

**Authors:** Kimberley J Anneveldt, Ingrid M Nijholt, Joke M Schutte, Jeroen R Dijkstra, Geert W J Frederix, Erwin Ista, Inez M Verpalen, Sebastiaan Veersema, Judith A F Huirne, Wouter J K Hehenkamp, Martijn F Boomsma

**Affiliations:** 1 Department of Radiology Isala Hospital Zwolle Netherlands; 2 Department of Gynecology Isala Hospital Zwolle Netherlands; 3 Julius Center for Health Science and Primary Care University Medical Center Utrecht Utrecht Netherlands; 4 Department of Internal Medicine, Section of Nursing Science Erasmus MC, University Medical Center Rotterdam Rotterdam Netherlands; 5 Department of Radiology Amsterdam University Medical Center Academic Medical Center Amsterdam Netherlands; 6 Department of Reproductive Medicine and Gynecology University Medical Center Utrecht Utrecht Netherlands; 7 Department of Gynecology Amsterdam University Medical Center Academic Medical Center Amsterdam Netherlands; 8 Department of Gynecology Amsterdam University Medical Center VU University Medical Center Amsterdam Netherlands

**Keywords:** high-intensity–focused ultrasound ablation, magnetic resonance imaging, interventional, leiomyoma, randomized controlled trial, cost-effectiveness analysis, clinical trial protocol

## Abstract

**Background:**

Magnetic resonance image–guided high-intensity–focused ultrasound (MR-HIFU) is a rather new, noninvasive option for the treatment of uterine fibroids. It is safe, effective, and has a very short recovery time. However, a lack of prospectively collected data on long-term (cost-)effectiveness of the MR-HIFU treatment compared with standard uterine fibroid care prevents the MR-HIFU treatment from being reimbursed for this indication. Therefore, at this point, when conservative treatment for uterine fibroid symptoms has failed or is not accepted by patients, standard care includes the more invasive treatments hysterectomy, myomectomy, and uterine artery embolization (UAE). Primary outcomes of currently available data on MR-HIFU treatment often consist of technical outcomes, instead of patient-centered outcomes such as quality of life (QoL), and do not include the use of the latest equipment or most up-to-date treatment strategies. Moreover, data on cost-effectiveness are rare and seldom include data on a societal level such as productivity loss or use of painkillers. Because of the lack of reimbursement, broad clinical implementation has not taken place, nor is the proper role of MR-HIFU in uterine fibroid care sufficiently clear.

**Objective:**

The objective of our study is to determine the long-term (cost-)effectiveness of MR-HIFU compared with standard (minimally) invasive fibroid treatments.

**Methods:**

The MYCHOICE study is a national, multicenter, open randomized controlled trial with randomization in a 2:1 ratio to MR-HIFU or standard care including hysterectomy, myomectomy, and UAE. The sample size is 240 patients in total. Women are included when they are 18 years or older, in premenopausal stage, diagnosed with symptomatic uterine fibroids, conservative treatment has failed or is not accepted, and eligible for MR-HIFU. Primary outcomes of the study are QoL 24 months after treatment and costs of treatment including direct health care costs, loss of productivity, and patient costs.

**Results:**

Inclusion for the MYCHOICE study started in November 2020 and enrollment will continue until 2024. Data collection is expected to be completed in 2026.

**Conclusions:**

By collecting data on the long-term (cost-)effectiveness of the MR-HIFU treatment in comparison to current standard fibroid care, we provide currently unavailable evidence about the proper place of MR-HIFU in the fibroid treatment spectrum. This will also facilitate reimbursement and inclusion of MR-HIFU in (inter)national uterine fibroid care guidelines.

**Trial Registration:**

Netherlands Trial Register NL8863; https://www.trialregister.nl/trial/8863

**International Registered Report Identifier (IRRID):**

DERR1-10.2196/29467

## Introduction

Fibroids are the most common benign gynecological tumors in women of reproductive age, occurring in up to 70% of the population. Approximately 25% of the uterine fibroids are symptomatic [1]. Symptoms include abdominal pain, menstrual disorders, lower urinary tract or bowel symptoms, and fertility disorders [2]. On a global level, fibroids represent an enormous economic burden to the health care system and costs can reach as much as US $5.9-34.4 billion each year in the United States [3]. Conservative treatment of fibroids fails in 50% of patients, many of whom subsequently opt for surgical procedures [4]. Hysterectomy is currently the most common treatment for symptomatic uterine fibroids, with millions of procedures performed annually around the world [5]. However, hysterectomies and myomectomies have a high risk of complications, long recovery, and might compromise future pregnancies [6], with the latter mainly due to peritoneal and intrauterine adhesions, a high rate of abnormal placentation, and fragility of myometrium as a result of myomectomy [7]. Furthermore, even a hysterectomy does not guarantee an intervention-free life, mostly because of complications caused by the operation itself [8]. This has led to a strong desire for less invasive treatments [4].

Currently, uterine artery embolization (UAE) is the only reimbursed minimally invasive treatment available in the Netherlands. The general treatment results after UAE are 60% fibroid volume reduction and on average 80%-90% patient satisfaction [9]. Complications after UAE include nontarget embolization, infection/septicemia and ovarian failure due to impairment of ovarian blood flow, and infection leading to fallopian tube damage with subsequent infertility [9,10].

Magnetic resonance image–guided high-intensity–focused ultrasound (MR-HIFU) is a thermal ablation technique, which enables noninvasive treatment of symptomatic uterine fibroids by selective tissue heating [11]. The ultrasound transducer produces convergent high-intensity ultrasound waves. The targeted tissue absorbs the acoustic energy leading to a temperature rise, which causes coagulative necrosis [12]. Magnetic resonance imaging (MRI) facilitates treatment planning and real-time monitoring by temperature mapping [13]. Directly after MR-HIFU, a contrast-enhanced MRI scan can visualize the ablated tissue, referred to as the nonperfused volume (NPV). NPV% (NPV divided by the initial fibroid volume) is one of the commonly used parameters to indicate technical treatment success [11].

When the MR-HIFU therapy of uterine fibroids was first introduced in clinical practice, it was allowed to ablate only 33%, and later on 50%, of the uterine fibroid. However, it soon became clear that clinical outcomes are closely related to high NPV percentages. Therefore, nowadays full ablation protocols are used [12,14]. In addition, better results and less adverse events were seen when using the latest generation of treatment devices [11]. Not all patients with symptomatic uterine fibroid are eligible for MR-HIFU treatment due to either patient or fibroid tissue characteristics, such as the number of fibroids or the extent of vascularization of a fibroid and the possibility to heat the tissue [15]. A wish to conceive is not a contraindication, although data on pregnancy outcomes remain sparse [16,17]. Careful screening is in all cases recommended [18]. Hitherto, only 5 studies were published on the cost-effectiveness of the MR-HIFU uterine fibroid treatment [19-23]. All used outdated, less effective MR-HIFU treatment protocols and costs of sanitary products, over-the-counter remedies, and alternative and complementary therapies were typically not taken into account. Nevertheless, these cost-effectiveness studies still concluded that MR-HIFU can be cost-effective at commonly accepted willingness-to-pay thresholds [11].

At this point, phase 1, 2a, and 2b studies according to the Idea, Development, Exploration, Assessment and Long-term study (IDEAL) framework have been completed in numerous sites all over the world [24], confirming safety and short- to middle-term technical and clinical outcomes. Conversely, no (non)randomized controlled trials are available in which MR-HIFU is directly compared with the current standard of care, and in which the full ablation protocol or the latest version of the MR-HIFU equipment was used. For example, in a comprehensive cohort trial comparing MR-HIFU with UAE, lower reintervention rates and greater improvement in symptoms were observed after UAE [25]. However, these results could be explained by impairment of ovarian reserve at follow-up in the UAE group and the use of outdated MR-HIFU equipment, which resulted in a rather low average NPV of 42.9% after treatment. With regard to follow-up, only 2 single-arm studies [26,27] with a follow-up of more than 12 months and using a full ablation protocol have been performed until now [11].

Because of the lack of randomized controlled trials (RCTs) that established long-term treatment outcomes and cost-effectiveness of MR-HIFU using an unrestricted, full ablation protocol and the latest equipment, we are now embarking on phase 3 of the IDEAL framework and will perform a randomized controlled (cost-)effectiveness study with a long-term follow-up.

The primary aim of this MYCHOICE study is to compare quality of life (QoL) at 24 months after MR-HIFU with QoL 24 months after standard fibroid care, which consists of hysterectomy, myomectomy, and UAE. Furthermore, we aim to determine the long-term cost-effectiveness of MR-HIFU compared with standard (minimally) invasive fibroid care. We expect that QoL after MR-HIFU is noninferior to QoL after standard care and that MR-HIFU is cost-effective compared with standard care.

## Methods

This protocol was developed according to the Standard Protocol Items: Recommendations for Interventional Trials (SPIRIT) 2013 statement [28].

### Study Design and Setting

The MYCHOICE study (MYoma treatment Comparison study: High-intensity image–guided fOcused ultrasound versus standard [minimally] Invasive fibroid care—a (Cost-)Effectiveness analysis; Netherlands Trial Register NL8863) is designed as an open, national, multicenter, RCT. By including both academic and nonacademic centers as participating hospitals in the MYCHOICE study, high volume and expertise are warranted. Participating hospitals provide a representative geographic spread across the country. All participating hospitals are specialized uterine fibroid centers and perform standard (minimally) invasive fibroid care. The MR-HIFU treatment will, however, be performed in the only 2 hospitals in the Netherlands that offer MR-HIFU treatment (Isala Zwolle and University Medical Center Utrecht) in addition to standard uterine fibroid care.

### Study Population and Eligibility

#### Overview

Our study population consists of women in the premenopausal phase visiting the gynecological outpatient clinic with symptoms caused by uterine fibroids. Symptoms of fibroids may comprise heavy menstrual bleeding and bulk symptoms such as pelvic pressure, micturition/defecation problems, or pain symptoms. A combination of several symptoms or a single symptom will be equally qualified as “symptomatic.” To optimize external validity of our study results, the inclusion and exclusion criteria defined in this study (Textbox 1) are similar to the inclusion and exclusion criteria applied for MR-HIFU in clinical practice. However, 2 exceptions are made. Women need to be motivated to undergo 1 of the 3 treatments in the control group, in case of being randomized to the control group, before participating in the MYCHOICE study. Furthermore, a wish to conceive within 1 year after inclusion is a reason to be not eligible for participating, because there is not yet a consensus about the standard of care for these women. Women without an active child wish but for whom a pregnancy in the future is not ruled out can be included in the study.

**Figure 1:**
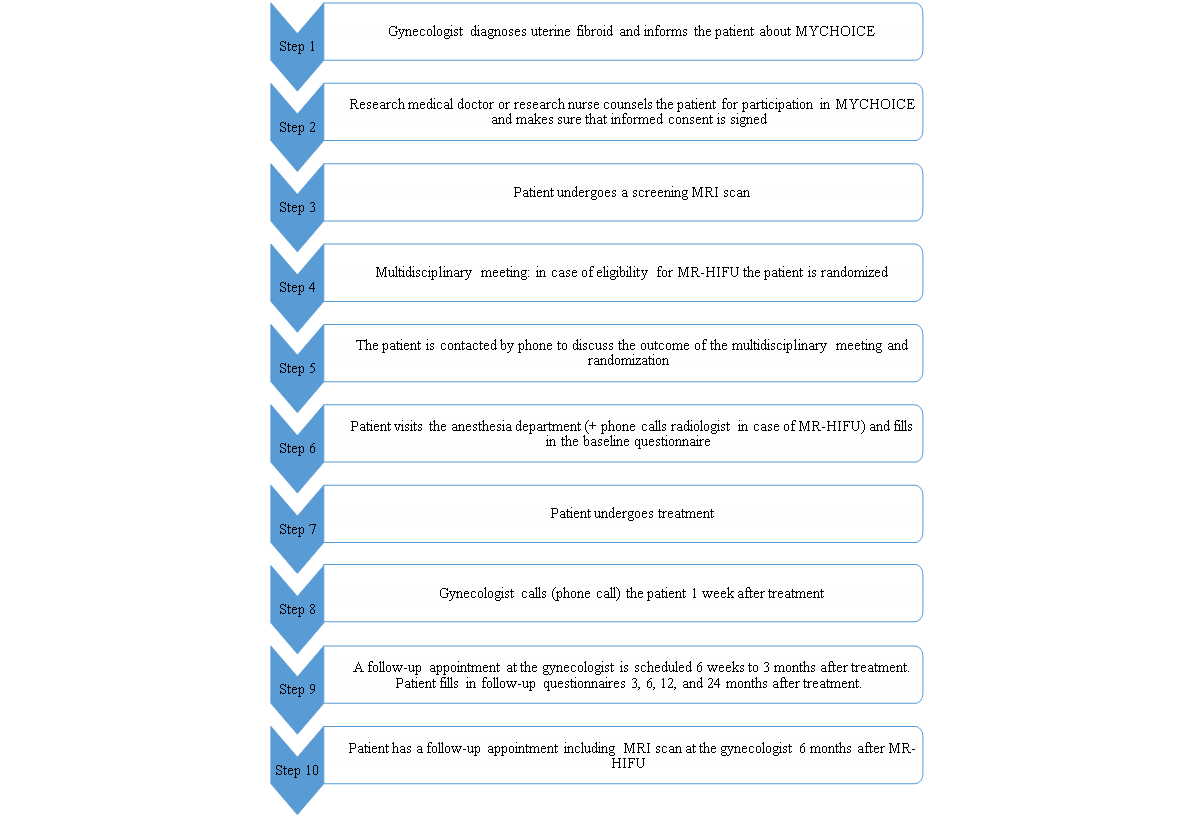
Flow diagram of the MYCHOICE procedure. MR-HIFU: magnetic resonance image–guided high-intensity focused ultrasound; MRI: magnetic resonance imaging; MYCHOICE: MYoma treatment Comparison study: High-intensity image–guided fOcused ultrasound versus standard (minimally) Invasive fibroid care—a (Cost-)Effectiveness analysis.

The MYCHOICE study procedure consists of several steps (Figure 1).

The eligibility procedure for this study consists of 2 screening phases. Only women that are considered eligible for participating in the study based on these 2 screening phases will be randomized.

**Inclusion criteria for participation in the MYCHOICE (MYoma treatment Comparison study: High-intensity image–guided fOcused ultrasound versus standard [minimally] Invasive fibroid care—a [Cost] Effectiveness analysis) study.**

**Inclusion criteria**

- Symptomatic fibroids warranting (minimally) invasive treatment, that is, either hysterectomy, myomectomy, or uterine artery embolization
- Conservative treatment failed or not accepted
- Premenopausal
- Age ≥18 years
- Eligible for magnetic resonance image–guided high-intensity focused ultrasound (MR-HIFU) treatment.

**Exclusion criteria**

- Asymptomatic fibroids
- Postmenopausal
- BMI of ≥35 kg/m^2^ or abdominal subcutis ≥4 cm or both
- More than 5 uterine fibroids unless 1 or 2 fibroids causing the symptoms can be clearly identified
- Magnetic resonance imaging contraindications or contrast allergy
- Current pregnancy
- A wish to conceive within 1 year after inclusion
- Suspicion of malignancy
- Dominant adenomyosis, defined as more volume of adenomyosis rather than fibroids
- Not willing to accept pretreatment with leuprorelin before MR-HIFU in case of a uterine fibroid with a diameter >10 cm or classified as Funaki 3
- Not willing to remove an interfering intrauterine contraception device prior to MR-HIFU
- Not eligible for MR-HIFU as determined by the multidisciplinary MR-HIFU team in Isala based on a screening magnetic resonance imaging:
  Uterine fibroid(s) either submucosal or subserosal stalked or with a diameter <2 cm
  Fibroids suitable for hysteroscopical removal
  Distance of abdominal wall to the dorsal side of uterine fibroids expected to be >12 cm even after the use of manipulation techniques
  Calcified uterine fibroids or fibroids without contrast enhancement

#### Phase 1 of the Screening Procedure

Patients presenting with uterine fibroid–related symptoms at the Department of Gynecology of the participating centers will undergo standard consultation, physical examination, and vaginal ultrasonography. The patient is briefly informed about the study when she appears to be eligible for participation in the study based on the physical examination and the vaginal ultrasonography (step 1 in Figure 1). In case the patient is interested in participating in the study, an appointment with a member of the research team or local research nurse will be made and the patient will receive more detailed study information to read at home (step 2 in Figure 1). In case a patient does not want to participate, the gynecologist asks the patient if she is willing to disclose the reason for not participating. During the appointment with a member of the research team or local research nurse, additional counseling will take place.

#### Phase 2 of the Screening Procedure

Once the patient has signed the informed consent form, a screening MRI scan according to a predefined protocol will be planned in the local hospital (step 3 in Figure 1).

Final eligibility of the patients of all participating centers will be determined by the multidisciplinary MR-HIFU team in the coordinating center based on the screening MRI scan and the inclusion and exclusion criteria (step 4 in Figure 1). These meetings will be accessible to members of the other participating hospitals. By performing central screening, a bias caused by differences per site is minimized and eligibility for MR-HIFU is secured.

### Intervention

#### Pretreatment

The participant’s gynecologist and general practitioner are informed about the outcome of the eligibility assessment and, if the patient is considered eligible, the randomization outcome (step 5 in Figure 1). Subsequently, the gynecologist will inform the patient about the outcome and baseline data will be collected by a member of the research team or the local research nurse and entered into the electronic case report form (Research Manager). In case a patient is randomized to the MR-HIFU treatment arm, she will be referred to an MR-HIFU performing hospital if her hospital is not 1 of the 2 hospitals in which the MR-HIFU treatment is performed. In case she is randomized to the standard care treatment arm, she can be treated in her own hospital.

#### MR-HIFU

MR-HIFU will be performed by well-trained and experienced radiologists using the latest version of the CE-marked Sonalleve MR-HIFU platform (Profound Medical Inc.) integrated into a 1.5-T MR-scanner (Achieva; Philips Healthcare) using a full ablation protocol. A uniform treatment protocol will be used in accordance with the manufacturer’s guidelines on the use of the device and the latest insights in the field of MR-HIFU treatment of uterine fibroids. Six months after treatment, a follow-up MRI scan will be performed before the follow-up appointment at the gynecologist (step 10 in Figure 1).

#### Control Group

The care as usual group will be offered surgery or UAE. Surgery will be either hysterectomy or myomectomy. Both hysterectomies and myomectomies can be performed by laparoscopy or laparotomy depending on the size and location of the fibroids. Participants allocated to the control group can decide together with their gynecologist which of the (minimally) invasive treatments they wish to undergo. All of the usual care treatments are performed extensively at the participating centers and will be performed according to national guidelines and local protocols. Surgery is preceded by a preoperative screening for anesthetic risk assessment. Depending on the modus of the hysterectomy or myomectomy, patients will be hospitalized for a minimum of 1-3 nights. UAE will be performed by well-trained and experienced radiologists. UAE can be either unilateral or bilateral. The patient usually has to stay in the hospital for 1-3 nights for careful pain monitoring after the procedure. Six weeks after all usual care treatments, a follow-up appointment at the gynecology department will be scheduled.

#### Use of Co-interventions

All included treatments aim for complete symptom reduction; however, clinical practice shows that additional treatment can be necessary during, for example, menstruation. Women can choose to use additional over-the-counter pain medication or prescribed medication such as oral contraception pills or antifibrinolytic drugs. These pills can influence symptom severity (both bleeding and pain). Therefore, data on the use of this medication are collected at both baseline and follow-up as part of the patient characteristics and medical consumption questionnaires.

### Data Collection

Data collection will take place before treatment, and at 3, 6, 12, and 24 months after treatment by questionnaires. Furthermore, baseline data of patient and treatment costs will be collected before treatment and after data lock-in (Table 1).

**Table 1:** Timeline of data collection.

Data	Baseline	Treatment	3 months	6 months	12 months	24 months	Data lock-in
Informed consent	X						
Patient characteristics^a^	X						
Pregnancy outcomes^b^	X					X	
UFS-QoL^b,c^	X		X	X	X	X	
EQ-5D-5L^b,d^	X		X	X	X	X	
Onset of menopause^b^						X	
(Time to) reintervention^a^			X	X	X	X	X
Adverse events/complications^a^		X	X	X	X	X	
PREM^b,e^	X		X	X	X	X	
Recovery time^a^		X	X	X			
Medical Consumption Questionnaire^b,f^	X		X	X	X	X	
Productivity Costs Questionnaire^b,f^	X		X	X	X	X	
Costs of treatment^a^							X
Reason for not participating^a,g^	X						

^a^Retrieved from questionnaires and medical record.

^b^Retrieved from questionnaires solely.

^c^UFS-QoL: Uterine Fibroid Symptom and Quality of Life questionnaire.

^d^EQ-5D-5L: 5-level version of the EuroQoL Questionnaire.

^e^PREM: patient-reported experience measurement

^f^Used for the cost-effectiveness analysis.

^g^Data collected by the gynecologist in case of not willing to participate.

### Outcomes

#### Primary Outcomes

In the MYCHOICE study, primary outcomes include (1) QoL at the follow-up time point of 24 months after treatment and (2) cost-effectiveness of MR-HIFU.

QoL is commonly measured with the validated Uterine Fibroid Symptom and Quality of Life questionnaire (UFS-QoL) [29]. This questionnaire consists of 2 parts: 8 symptom questions and 29 questions concerning health-related QoL with 6 subscales. The 8 symptom severity questions concern duration, frequency and severity of menstruation, urination pattern, tightness or pressure in the pelvic area, and fatigue. The 6 subscales of the HR QoL part of the questionnaire are concern, activities, energy/mood, control, self-consciousness, and sexual function. All items are scored on a 5-point Likert scale. Both internal consistency reliability (subscale Cronbach α=.83-.95, overall health-related QoL score α=.97) and test–retest reliability (intraclass correlation coefficients 0.76–0.93) of this questionnaire were shown to be adequate. Moreover, the UFS-QoL has an excellent construct and discriminative validity [29]. From the symptom-specific part of the questionnaire, a symptom severity score (SSS; range 0-100, with higher scores indicating more [severe] symptoms) can be calculated. Because symptom reduction is the main aim of all uterine fibroid treatments, we define QoL at the follow-up time point of 24 months as a change in reported symptom severity compared with baseline.

The cost-effectiveness analysis will be performed from a societal perspective. Cost-effectiveness will be reported as incremental cost-effectiveness ratio, that is, the ratio between the expected difference in cost and the expected difference in effect (clinical effect or utility [quality-adjusted life year] and net [monetary] benefit). Cost-effectiveness acceptability curves will be presented to summarize the impact of uncertainty on the result of the economic evaluation.

The Dutch value set will be applied to the 5-level version of the EuroQoL questionnaire (EQ-5D-5L) to produce quality-adjusted life year values [30].

We consider 4 cost categories: (1) direct medical in-hospital costs (eg, preprocedural costs, in-hospital costs related to the intervention, any additional in-hospital medical costs during follow-up); (2) direct medical out-of-hospital costs (eg, unscheduled general practitioner visits and use of medication out of hospital); (3) direct nonmedical costs (patient expenses such as travel costs and sanitary measures); and (4) indirect costs (productivity-related costs due to absence from work) [31].

The unit costs of direct medical in-hospital cost volumes will be based on Dutch guidelines for economic evaluations. The cost volumes of MR-HIFU, UAE, myomectomy, and hysterectomy are based on detailed microcosting by using data recorded in the case record forms and patient records in all participating hospitals. The cost volumes related to complications will be recorded prospectively in the case record form (eg, type of complication, unscheduled outpatient visit, subsequent diagnostic and therapeutic measures). All interventions include 1 follow-up by phone (at 1 week after primary intervention; Figure 1). In case of an UAE, myomectomy, and hysterectomy, 1 follow-up visit at the outpatient gynecology department will be planned at 6 weeks after the primary intervention; in case of MR-HIFU at 3 and 6 months, a follow-up appointment at the gynecology department will take place, at 6 months combined with an MRI scan. This will be considered standard care, and will therefore be included in our cost analysis. Any further follow-up visits conducted for study purposes will be excluded from our analysis unless these are unscheduled follow-up visits for medical problems related to the primary intervention.

The unit costs of direct medical out-of-hospital costs, direct nonmedical costs, and indirect costs will also be based on Dutch guidelines for cost calculations in health care. The following altered patient questionnaires will be used: iMTA Productivity Cost Questionnaire (iPCQ) and iMTA Medical Consumption Questionnaire (iMCQ). The iPCQ questionnaire is a short generic measurement instrument on the impact of disease on the ability of a person to perform work. It also contains questions about absence from unpaid labor. This questionnaire is a generic instrument for measuring medical costs, including questions related to frequently occurring contacts with health care providers. All questionnaires will be sent by email or post according to the preference of the participant at baseline and at 3, 6, 12, and 24 months after treatment (Table 1). Patients will receive an automatic reminder by email. Indirect costs due to absence from work will be estimated as the actual working time lost (hours) multiplied by the average net income according to the friction cost method.

A decision analytic model with lifetime horizon will be developed by combining costs and effects. Complete uncertainty analyses (deterministic and probabilistic sensitivity analyses) will be performed.

In addition, a budget impact model will be constructed, taking the (gradual) implementation of MR-HIFU over time, the initial investments, and the savings into account that were shown to be realistic in the trial. The model will use different perspectives: (1) The net Dutch Budgetary Framework for Healthcare (Budgettair Kader Zorg) perspective; and (2) health insurance/third-party payer perspective.

The budget impact model is performed through modeling and analyzed in a probabilistic way.

Because MR-HIFU is an outpatient treatment with a fast(er) recovery, it is expected to be cheaper than the current standard (minimally) invasive treatments, especially from a societal perspective.

#### Secondary Outcomes

Data on several secondary outcomes will be collected (Table 2). These include adverse events and complications during treatment and recovery, cost-effectiveness–relevant outcomes such as hospital stay duration and use of (co)medication, patient-reported experiences, reintervention rate in case a uterine-sparing treatment was performed, reproductive outcomes when applicable, and technical outcomes after MR-HIFU, such as NPV reached.

**Table 2:** Secondary outcomes of the MYCHOICE^a^ study, including measurement tool and statistical analyses.

Outcome	Description	Measurement	Statistical analyses
QoL^b^ parameters	QoL is expected to be negatively correlated with symptom severity. When symptoms decrease, the QoL is expected to improve.	UFS-QoL^c^ questionnaire and EQ-5D-5L^d^ questionnaire.	The time course of the change in health-related quality of life after treatment will be analyzed using longitudinal covariance analysis similar to the analysis of the change in SSS^e^.
Adverse events and complications	The nature of adverse events and complications of the 2 treatment arms are expected to differ.	Adverse events will be classified according to the classification of surgical complications [32].	Adverse events are analyzed using descriptive statistics. Adverse events per treatment group and treatment will be presented with their occurrence rate.
Length of hospital stay	Reduced hospital stay is beneficial in terms of health care costs and is also considered as a great advantage by patients.	Length of hospital stay will be collected from the patient hospital file.	The average length of the hospital stay will be reported as mean (SD) or median (interquartile range).
Periprocedural and postprocedural pain	Pain perception may influence treatment experience and therefore satisfaction with the treatment.	Periprocedural and postprocedural pain will be measured on a numerical rating scale from 0 to 10 in the 3 months’ follow-up questionnaire. Pain complaints at 3, 6, 12, and 24 months after treatment will be registered by the amount and duration of pain killers used.	The numerical rating score is considered to be a semicontinuous measure (range 0-10: higher score represents more pain). Pain experienced will be reported as mean (SD) or median interquartile range.
Patient-reported satisfaction with treatment and treatment preference (PREM^f^)	Because a randomized controlled trial will be performed, satisfaction with treatment might be affected by not being allocated to preferred treatment. Furthermore, we expect women declining participation because of the randomization aspect of the trial.	The PREM consists of a concise set of statements about the experience of the patients with the treatment and whether they would recommend the treatment to a friend. In addition, the preference will be registered for a particular treatment of potential participants before randomization. Women who decline participation in the study will also be asked if they are willing to disclose their reasons for declining.	The PREM score is scored on a 5-point Likert scale: higher score represents better experience. Whether there is a difference in PREM outcome between the 2 treatment arms is determined by linear regression analysis.
(Co)medication	Women (still) experiencing symptoms after treatment may take or may start to take medication to relieve these symptoms. Medication might also be used as contraceptive and to mask possible fibroid-related symptoms at the same time.	Data on any prescribed or over-the-counter medication taken to reduce fibroid symptoms as reported by the patient will be collected via the questionnaires at baseline, 3, 6, 12, and 24 months after treatment.	The number of women taking medication to suppress fibroid-related symptoms is measured at baseline and 3, 6, 12, and 24 months after treatment. The absolute numbers and percentage of women taking comedication per group per time point will be presented.
Reintervention rate and time to reintervention	A reintervention is defined as an additional intervention due to persisting or recurring symptoms of the treated fibroid or due to complications of the initial fibroid treatment.	Occurrence and type of reintervention are collected via both electronic patient file and questionnaires at 3-, 6-, 12-, and 24-month follow-up.	The reintervention rate at the follow-up time point of 24 months after MR-HIFU^g^ is presented as percentage reinterventions with its 95% CI. Reintervention rate will be presented per treatment arm but also per treatment. To investigate whether the time to reintervention differs between the 2 treatment arms, Cox proportional hazards analysis will be used.
Onset of menopause after uterus-saving treatments	Uterine fibroid symptoms diminish after menopause along with fibroid-related symptoms. Because this may affect symptom reduction, QoL, and the possible need for a reintervention, the menopausal state of the participants will be determined.	Onset of menopause is defined as 1 year without menstrual bleeding and measured by a questionnaire at the 24-month follow-up.	The absolute numbers and percentage of postmenopausal women per group and treatment will be presented.
Reproductive outcomes after uterus-saving treatments	Only women with an active wish to conceive within 1 year after treatment will be excluded from this study. Thus, some women may get pregnant after the uterus-saving treatment.	Reproductive outcomes will be collected of all women that underwent a uterus-saving treatment by a questionnaire at 24-month follow-up.	Reproductive outcomes will be presented per uterus-saving group using absolute numbers and percentage.
NPV^h^ and fibroid shrinkage after MR-HIFU treatment	Technical success of MR-HIFU is commonly presented as NPV percentage directly after treatment or fibroid shrinkage 6 months after treatment, as determined on an MRI^i^ scan.	NPV% will be measured on an MRI scan performed directly after treatment. Fibroid shrinkage will be measured by comparing volume measured on 6 months’ follow-up MRI scan with pretreatment volume.	We will investigate whether technical success (NPV% [NPV/initial volume of the fibroid] or fibroid shrinkage) is associated with (long-term) effectiveness using regression analysis.
Other study parameters	Several patient characteristics are collected from the medical record of the patients and from the baseline questionnaire such as age, amount and size of fibroids, location of fibroid, position of uterus, duration of treatment, ethnicity, parity, height, weight, relevant medical, and medical history.	Data analysis will be stratified by center to check for differences in results between centers.	In case necessary, multilevel analysis will be used to correct for differences between centers. Multivariate analysis will be performed for symptom reduction, QoL improvement, and reintervention correcting for comedication or menopause as possible confounder. In addition, we will investigate whether certain baseline characteristics such as age, BMI, number of uterine fibroids, and target fibroid size are associated with symptom reduction and reintervention using linear, logistic, and Cox regression analysis.

^a^MYCHOICE: MYoma treatment Comparison study: High-intensity image–guided fOcused ultrasound versus standard (minimally) Invasive fibroid care—a (Cost-)Effectiveness analysis.

^b^QoL: quality of life.

^c^UFS-QoL: Uterine Fibroid Symptom and Quality of Life questionnaire.

^d^EQ-5D-5L: 5-level version of the EuroQoL questionnaire.

^e^SSS: symptom severity score.

^f^PREM: patient-reported experience measurement.

^g^MR-HIFU: magnetic resonance image–guided high-intensity focused ultrasound.

^h^NPV: nonperfused volume.

^i^MRI: magnetic resonance imaging.

### Sample Size

The MYCHOICE study is a noninferiority trial for which we hypothesize that MR-HIFU is noninferior to the group of standard (minimally) invasive treatments, accepted by a ≤15 points difference in symptom reduction at 24 months’ follow-up as determined with the SSS (range 0-100 points) part of the UFS-QoL questionnaire. We expect that women participating in this trial have a slight preference for the noninvasive MR-HIFU treatment. We therefore choose to use an unbalanced design in which participants are allocated to the intervention or usual care group at a 2:1 ratio, resulting in a larger sample size in the MR-HIFU treatment group. With a larger sample size of the MR-HIFU treatment group, we will be able to gather more data on this new treatment while the effectiveness of standard care is already much better documented. Randomization, stratified by center, will be performed using a computer-generated randomization system, which randomly selects block sizes of 3, 6, or 9. Previous studies concerning (minimally) invasive treatments were performed with women with an average baseline UFS-QoL SSS of 55-65 points [33]. Treatment initiated a decrease of 30-47 points on SSS 12 months after these combined treatments. Hitherto, there are 2 MR-HIFU studies published that used a full ablation protocol, had the same 12-month follow-up period as the studies on (minimally) invasive treatments, and in which women participated with a baseline UFS-QoL SSS of 55-65 points. These women showed an SSS reduction of 30-40 points at follow-up [34,35]. In our study population we expect comparable baseline SSS in the MR-HIFU and standard care group. However, because hysterectomy results in a somewhat higher SSS reduction than the uterus-saving treatments, we assume in our power calculation an a priori 5-point delta between both the MR-HIFU and standard care group in favor of the standard care group. Using a noninferiority margin of 15 points with α (1-sided)=0.025, β=.1, and an SD of 20 points, we estimate that 192 participants (128 patients in the MR-HIFU treatment group and 64 patients in the [minimally] invasive treatment group) will be required to test noninferiority of MR-HIFU. Anticipating a 20% loss to follow-up, we need to include 160 patients in the MR-HIFU group and 80 patients in the (minimally) invasive treatment group. The noninferiority margin and the SD of 20 points were determined in consultation with the Dutch Society for Obstetrics and Gynecology and were similar to the noninferiority margin used in the MYOMEX-2 study [36].

### Recruitment

In all participating hospitals, patients will be recruited during a visit at the gynecologist. Furthermore, a study website is created to inform patients from all over the country, providing information and contact details to directly contact the study team. By promoting this website among general practitioners, gynecologists, and potential participants, we expect women with an interest in MR-HIFU to become acquainted with our study. Because MR-HIFU treatment for uterine fibroids is not reimbursed in the Netherlands, participating in the MYCHOICE study is the only possibility for women to undergo MR-HIFU treatment.

### Statistical Methods

In this study, data will initially be analyzed on an intention-to-treat-basis (including treatment failures) but a per-protocol analysis will also be performed. QoL at the follow-up time point of 24 months after treatment is determined as a change in SSS between baseline and 24 months’ follow-up. The difference between the symptom reduction after MR-HIFU and standard (minimally) invasive fibroid care including 97.5% CI is determined using linear regression analysis with a correction for baseline SSS. Although symptom reduction 24 months after MR-HIFU will be expected to be noninferior to standard (minimally) invasive fibroid care, the time course of symptom reduction may differ between the 2 treatment arms. This will be investigated with longitudinal covariance analysis. A faster symptom reduction in the usual care arm may be caused by a faster symptom reduction after hysterectomy. Therefore, a subgroup analysis in which the individual treatments are compared will be performed.

Patients can leave the study at any time for any reason if they wish to do so, without any consequences. In case they decide to withdraw before treatment or within the first 3-month follow-up, they will be included in the database, but an additional patient will be included to achieve the required sample size and reach primary outcome. In case patients withdraw after the 3-month follow-up, they are considered nonresponders. As much precautions as possible will be taken to prevent missing data. However, missing values are expected to occur in our trial due to technical failures and loss to follow-up. In case missing data reach 5%, additional analyzes will be performed to identify a plausible assumption, that is, missing not at random, missing at random, or missing completely at random. Subsequently, an analysis method that is valid under that assumption will be used.

### Data Monitoring

No data monitoring committee will be installed because the risks of participation in this study is categorized as insignificantly low. A data management plan is developed, detailing data management procedures, data standards, minimal data set requirements, and protocols (Isala Institutional Research Board). Data are collected in an online data management platform (Research Manager). Data will be securely stored for at least 15 years, according to hospital Institutional Research Board storage protocols. The study sponsor will be in charge of overseeing data management and access procedures. The Research Manager software will assign a “study ID.” The reference between the study ID and the hospital patient number is listed in the patient identification log. The patient identification log will only be accessible by authorized personnel. Each electronic case report form will be completed on-site by the investigator or an authorized staff member. All imaging data will be stored on location but transferred in preparation of the multidisciplinary meeting. After the multidisciplinary meeting these data will be destroyed for privacy reasons. All individual patient data records will be collected on a confidential basis and according to the applicable national data protection, privacy, and secrecy laws.

### Safety Reporting

Adverse events are defined as undesirable experiences of a participant within 30 days after treatment and related to participation in this study. All adverse events reported by the participant or observed by the investigator or study staff will be recorded. A serious adverse event is any untoward medical occurrence, within 30 days after treatment and related to participation in this study and results in death, is life threatening (at the time of the event), requires hospitalization or prolongation of existing inpatients’ hospitalization, results in persistent or significant disability or incapacity, or any other important medical event that did not result in any of the outcomes listed above. The investigator will report all serious adverse events to the sponsor without undue delay after obtaining knowledge of the events.

### Auditing

The clinical monitor will be responsible for verifying adherence to the protocol, reviewing participant records and source data, maintaining records of all actions taken to correct protocol deficiencies during the investigation, and assuring that the data needed to complete the study are complete and accurate.

### Patient and Public Involvement

The Foundation Bekkenbodem4All (Pelvicfloor4All) was consulted on the design of the study from a patient perspective and their opinion and feedback were taken into consideration. In addition, an evaluation meeting with previous MR-HIFU patients took place. Outcomes of this meeting were used to improve MR-HIFU treatment routine and to point out important patient outcomes. During inclusion, Bekkenbodem4All will promote the study via their network and participate in the yearly meetings in which the progression of the study is discussed. When the results of the study warrant uptake of the treatment in standard reimbursed care, they will aid in the final implementation of the treatment.

### Ethics Approval

This protocol, informed consents, and patient information have been approved by the local medical ethical committee of Isala Hospital (NL74716.075.20) on September 24, 2020, with respect to scientific content and compliance with applicable research and human patient regulations. The research activities of the MYCHOICE study comply with the international conventions and codes of conduct, and the latest Helsinki Declaration of the World Medical Association adopted by the World Medical Assembly.

### Dissemination Policy

We aim to make all data Findable, Accessible, Interoperable and Reusable (FAIR) according to the FAIR principles [37]. Therefore, we will assign all (meta)data with a unique and persistent (global) identifier and register or index them in a searchable digital data repository at the end of the study for long-time archiving and data reuse purposes. Results will be presented in (inter)national congresses and meetings, and will be published in peer-reviewed journals, publications of the patient associations, in health-related journals, and on various websites such as the MYCHOICE study website.

## Results

Inclusion for the MYCHOICE study started in November 2020. Patient enrollment is expected to last approximately 36 months. Because of the 24-month follow-up, we expect to complete data collection in 2026 and plan the dissemination of the results subsequently.

## Discussion

### Added Value of MYCHOICE

The MYCHOICE study distinguishes itself from previous MR-HIFU trials in that it is an RCT in which full ablation protocols and the latest MR-HIFU equipment are used for uterine fibroid treatment. Moreover, patient follow-up is 24 months. Furthermore, it answers important research questions on both effectiveness and cost-effectiveness with outcomes that are relevant for policy makers, physicians, and patients.

### Strengths

As a primary outcome, we will use QoL in terms of symptom reduction 24 months after treatment. We did not choose the commonly used outcome in uterine fibroids studies, reintervention rate, as our primary outcome because re-interventions are not expected to occur after hysterectomy. Symptom reduction will most likely also differ between hysterectomy and uterus-saving treatment options. However, the influence of symptom reduction after hysterectomies in the control group is probably limited, because we expect that most women who will participate in the MYCHOICE study prefer a uterus-preserving treatment option, just like the intervention under study. This is further enhanced by the 2:1 randomization ratio. This ratio will lead to a higher chance to undergo MR-HIFU treatment, and we believe is therefore an important strength of the design. Another strength is the fact that this unbalanced design will enable us to gather more data on our intervention, while the effectiveness of standard care is already much better documented.

Our follow-up duration of 24 months is based on the long-term outcomes of a retrospective study on MR-HIFU treatment results performed by our group [14]. In this study, we found that all reinterventions were performed within 24 months after the initial treatment, indicating that the treatment effect reaches a steady state within 24 months after treatment. Thus, it is not useful to prolong follow-up of these patients.

### Limitations

A possible limitation of the MYCHOICE study is the uncommon use of a mixed control group. However, in current daily practice, usual care for women with symptomatic uterine fibroids in whom conservative treatment failed or is undesired consist of several (minimally) invasive treatments. The minimally invasive UAE is reimbursed in the Netherlands, and would therefore be an appropriate reference treatment for the noninvasive MR-HIFU treatment. However, hysterectomies are by far the most frequently performed and should thus not be omitted from the standard care group. The standard care group is complemented with myomectomies. We expect that women willing to participate in this study are mostly searching for a uterus-saving treatment option, sometimes because of a future pregnancy wish. For this category of women, myomectomy is the only alternative and therefore a mixed control group qualifies the most. By using this mixed group, we believe we best represent the real-world situation. Furthermore, the information on treatment preference in the control group can be used to gain more insights into patient preferences.

Although an RCT design is commonly considered to provide the best evidence on the effectiveness of a new intervention compared with usual care, our RCT design also poses several challenges [38]. Women may not be willing to be randomized, which may delay enrollment, and our 2:1 randomization ratio with a mixed control group may lead to low sample sizes for the individual treatment options in the mixed control group, which will limit valid comparisons between outcomes of individual treatments. However, sufficient data on primary outcomes are already available for all treatments in this control group. Other possible limitations of the MYCHOICE study are that not all (secondary) outcomes are equally relevant for all included treatments and that the MR-HIFU treatment cannot be performed in all participating centers. However, because of the restricted number of patients eligible for treatment and the complexity of the treatment, it might not be cost efficient to have more than 2-4 uterine fibroid MR-HIFU facilities in the Netherlands.

## Abbreviations

EQ-5D-5L: 5-level version of the EuroQoL questionnaire
FAIR: Findable, Accessible, Interoperable and Reusable
IDEAL: Idea, Development, Exploration, Assessment and Long-term study
iMCQ: iMTA Medical Consumption Questionnaire
iPCQ: iMTA Productivity Cost Questionnaire
MR-HIFU: magnetic resonance image–guided high-intensity focused ultrasound
MRI: magnetic resonance imaging
MYCHOICE: MYoma treatment Comparison study: High-intensity image–guided fOcused ultrasound versus standard (minimally) Invasive fibroid care—a (Cost-)Effectiveness analysis
NPV: nonperfused volume
PREM: patient-reported experience measurement
QoL: quality of life
RCT: randomized controlled trial
SPIRIT: Standard Protocol Items: Recommendations for Interventional Trials
SSS: symptom severity score
UAE: uterine artery embolization
UFS-QoL: Uterine Fibroid Symptom and Quality of Life questionnaire
